# Atypical Hemolytic Uremic Syndrome With Hypocellular Bone Marrow: A Report of a Rare Case

**DOI:** 10.7759/cureus.111698

**Published:** 2026-06-29

**Authors:** Champai R Soren, Vibin K Vasudevan, Rachna Sharma, Naresh Lal

**Affiliations:** 1 Paediatrics, Kalinga Institute of Industrial Technology (KIIT), Bhubaneswar, IND; 2 Paediatrics, BLK-Max Super Speciality Hospital, New Delhi, IND

**Keywords:** ahus, aplastic anemia, bone marrow hypocellularity, plasma exchange, thrombotic angiopathy

## Abstract

Atypical hemolytic uremic syndrome (aHUS) is not a common thrombotic microangiopathy (TMA) characterized by microangiopathic hemolytic anemia, thrombocytopenia, and renal impairment, often due to dysregulation of the alternative complement pathway. Bone marrow study in HUS is classically hypercellular. However, hypo-cellularity is rarely encountered.

We report a case of a five-year-old boy who presented with severe pallor, thrombocytopenia, hypertension, and signs of TMA, including schistocytosis and renal impairment. Infectious and autoimmune causes were excluded. Initial management with plasma exchange and other supportive measures showed no improvement. ADAMTS13 activity was within normal limits, which excluded thrombotic thrombocytopenic purpura (TTP). Further investigations, including next-generation sequencing and anti-complement factor H (anti-CFH) antibody tests, were negative. To our surprise, bone marrow biopsy revealed markedly hypocellular marrow (10-15% cellularity), indicative of aplastic anemia. Despite escalation of therapy with steroids, intravenous immunoglobulin (IVIG), and eculizumab, the child showed no response and succumbed to a pulmonary hemorrhage.

The case highlights a rare and fatal presentation of aHUS associated with bone marrow hypoplasia, an uncommon and diagnostically challenging phenotype. It underscores the need for high clinical suspicion, early bone marrow evaluation in refractory cases, and the importance of timely access to complement-targeted therapies. The coexistence of aHUS and aplastic anemia suggests a potential novel pathophysiologic overlap warranting further investigation.

## Introduction

Thrombotic microangiopathies (TMAs) comprise a group of disorders characterized by microangiopathic hemolytic anemia, thrombocytopenia, and organ injury caused by widespread microvascular thrombosis, most frequently involving the kidneys. Thrombotic thrombocytopenic purpura (TTP) results from a congenital or acquired deficiency of ADAMTS13 and is often associated with neurological manifestations. In contrast, atypical hemolytic uremic syndrome (aHUS) develops because of inherited or acquired abnormalities affecting the regulation of the alternative complement pathway, leading to endothelial damage and TMA [1].

Bone marrow examination in patients with HUS is generally reported as hypercellular. To our knowledge, only a single case describing hypocellular marrow findings in association with HUS has been reported previously [2].

The diagnosis of aHUS remains challenging. The disease is rare, complement testing lacks optimal sensitivity and specificity, and approximately 30-50% of affected individuals do not demonstrate identifiable genetic or acquired complement abnormalities. Furthermore, TMA may occur in several complement-activating conditions, and no single diagnostic test definitively confirms aHUS. Consequently, the diagnosis is largely one of exclusion. Prompt recognition and treatment are essential because early intervention is associated with improved outcomes. Therefore, aHUS should be considered in any patient presenting with TMA [3].

We present a case of idiopathic aHUS associated with marked bone marrow hypoplasia consistent with aplastic anemia.

## Case presentation

A five-year-old boy presented with progressive pallor of two weeks’ duration, low-grade fever for two days, and a single episode of epistaxis. He had a known history of Grade I pilocytic astrocytoma located in the cerebellopontine angle, diagnosed at two years of age, accompanied by right-sided facial palsy. The tumor had remained stable on serial neuroimaging and was being managed conservatively. The family reported prior use of herbal and Ayurvedic medications, which had been discontinued one week before admission.

On examination, the child appeared irritable and distressed. He exhibited severe pallor, age-inappropriate tachycardia and tachypnea, and marked hypertension (180/110 mmHg, above the 99th percentile for age). Oxygen saturation was 98% on room air. Petechial lesions were present over the face, oral mucosa, and abdomen. Hepatomegaly was noted, with the liver palpable 4 cm below the right costal margin, while splenomegaly was absent. Neurological assessment demonstrated residual facial nerve palsy without additional deficits. Cardiovascular and respiratory examinations were otherwise unremarkable.

Peripheral blood smear showed features of microangiopathic hemolysis with numerous schistocytes. Bone marrow aspiration and biopsy unexpectedly demonstrated marked hypocellularity, with an estimated cellularity of 10-15%, consistent with aplastic anemia.

Laboratory investigations at admission are summarized in Table 1.

**Table 1:** Summary of laboratory investigations at admission *Pediatric reference ranges may vary slightly depending on age and laboratory standards. PNH: paroxysmal nocturnal hemoglobinuria; NGS: next-generation sequencing; anti-FCH Ab: anti-complement factor H antibody; ADAMTS 13: A disintegrin-like metalloproteinase with thrombospondin motif type 1 member 13; PT: prothrombin time; APTT: activated partial thromboplastin time; ESR: erythrocyte sedimentation rate; LDH: lactate dehydrogenase

Investigation	Result	Pediatric Reference Range*	Interpretation
Hemoglobin	8.3	11.0-14.5 g/dL	Low
Total Leukocyte Count (TLC)	3,700	5,000-15,000/cumm	Low
Neutrophils	28	40-70%	Low
Lymphocytes	68	20-50%	High
Absolute Neutrophil Count (ANC)	1,050	>1,500/µL	Mild neutropenia
Platelet Count	40,000	150,000-450,000/cumm	Severely low
ESR	38	0-10 mm/hr	High
Corrected Reticulocyte Count	2.7	0.5-2.0%	Mildly elevated
Blood Urea	43.8	7-20 mg/dL	High
Serum Creatinine	0.74	0.3-0.7 mg/dL	Mildly elevated
Baseline Creatinine	0.3	0.3-0.7 mg/dL	Normal baseline
LDH	587	100-300 U/L	High
Ferritin	608	7-140 ng/mL	High
Urine Protein	3+	Negative	Significant proteinuria
Urine RBC	10-12	0-2/HPF	Hematuria
Peripheral Smear	Microangiopathic anemia with schistocytes (6%)	No schistocytes	Abnormal
Bone Marrow Aspiration and Biopsy	Hypocellular marrow (10-15% cellularity)	Hypercellular marrow	Aplastic anemia
Schistocytes	6	<1%	Markedly elevated
Direct Coombs Test	Negative	Negative	Normal
PT	Normal	Normal range	Normal
APTT	Normal	Normal range	Normal
Fibrinogen	Normal	200-400 mg/dL	Normal
Serum Electrolytes	Normal	Within normal limits	Normal
Liver Function Tests	Normal	Within normal limits	Normal
Blood Culture	Negative	Negative	Normal
Urine Culture	Negative	Negative	Normal
Malaria Workup	Negative	Negative	Normal
Dengue Workup	Negative	Negative	Normal
Scrub Typhus Workup	Negative	Negative	Normal
Leptospira Workup	Negative	Negative	Normal
Autoimmune Workup	Negative	Negative	Normal
Parvo B19 Virus	Negative	Negative	Normal
Flow Cytometry for PNH	Negative	Negative	Normal
NGS	Negative	Negative	Normal
Anti-FCH Ab	Negative	Negative	Normal
ADAMTS 13 Activity	40% Activity	50-150%	Mildly reduced activity

**Figure 1:**
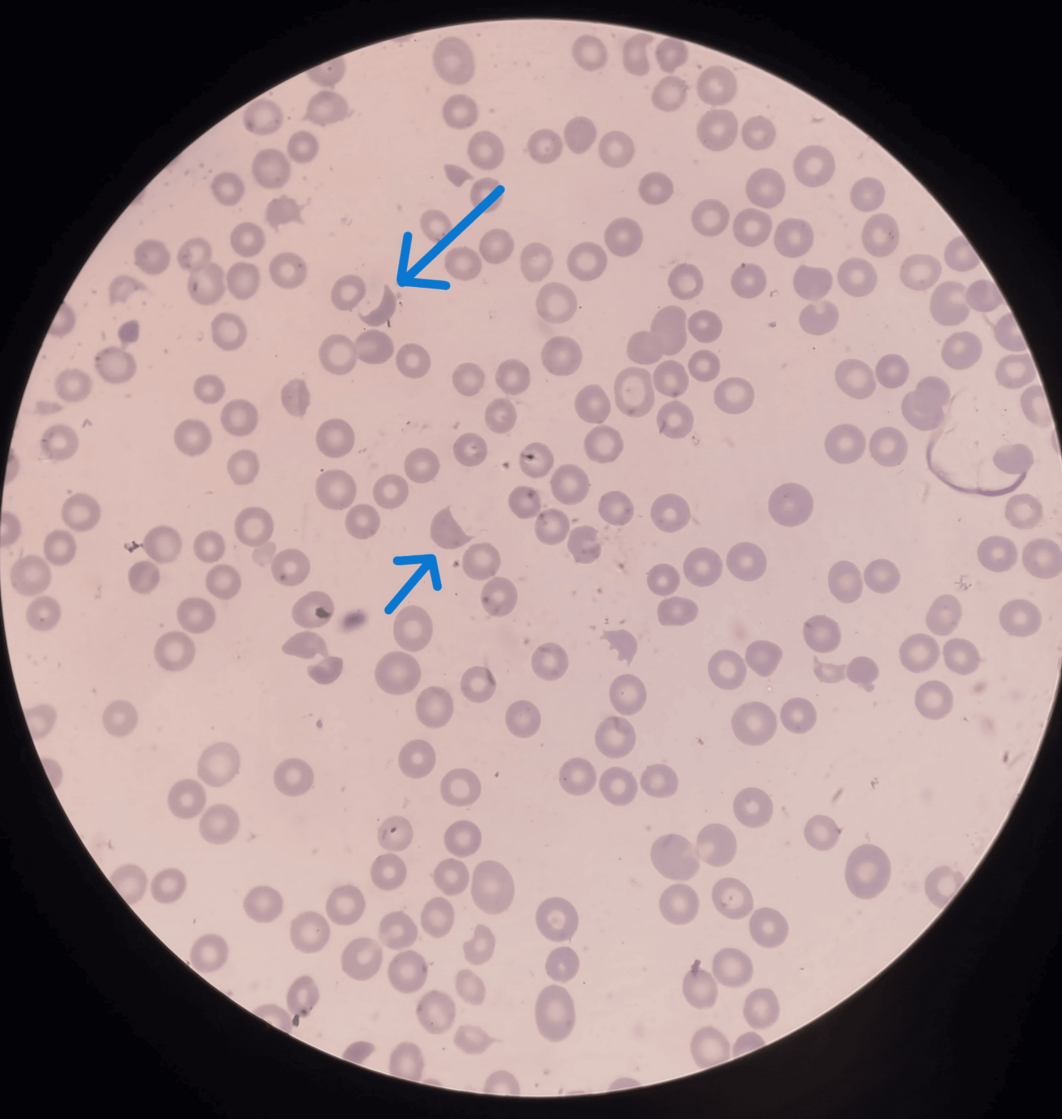
Peripheral blood film showing numerous schistocytes (blue arrows), suggestive of microangiopathic hemolysis (Giemsa stain, ×400)

Peripheral smear examination revealed microangiopathic anemia with leukopenia, thrombocytopenia, and schistocytosis (6%) (Figure 1).

**Figure 2:**
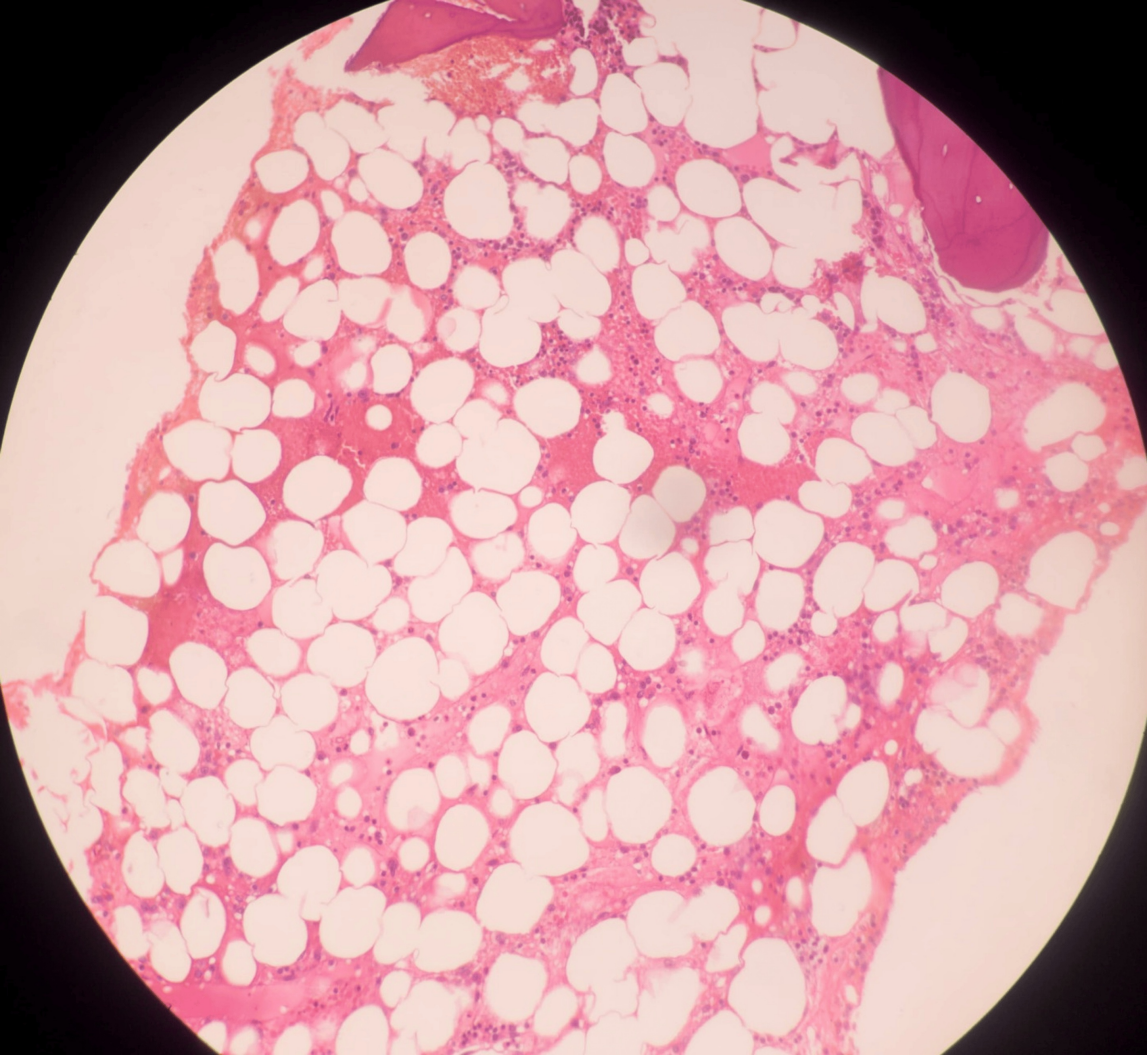
Hypocellular bone marrow biopsy (10%-15% cellularity) showing predominantly lymphocytes, plasma cells, and histiocytes (hematoxylin and eosin stain, ×200 magnification)

**Figure 3:**
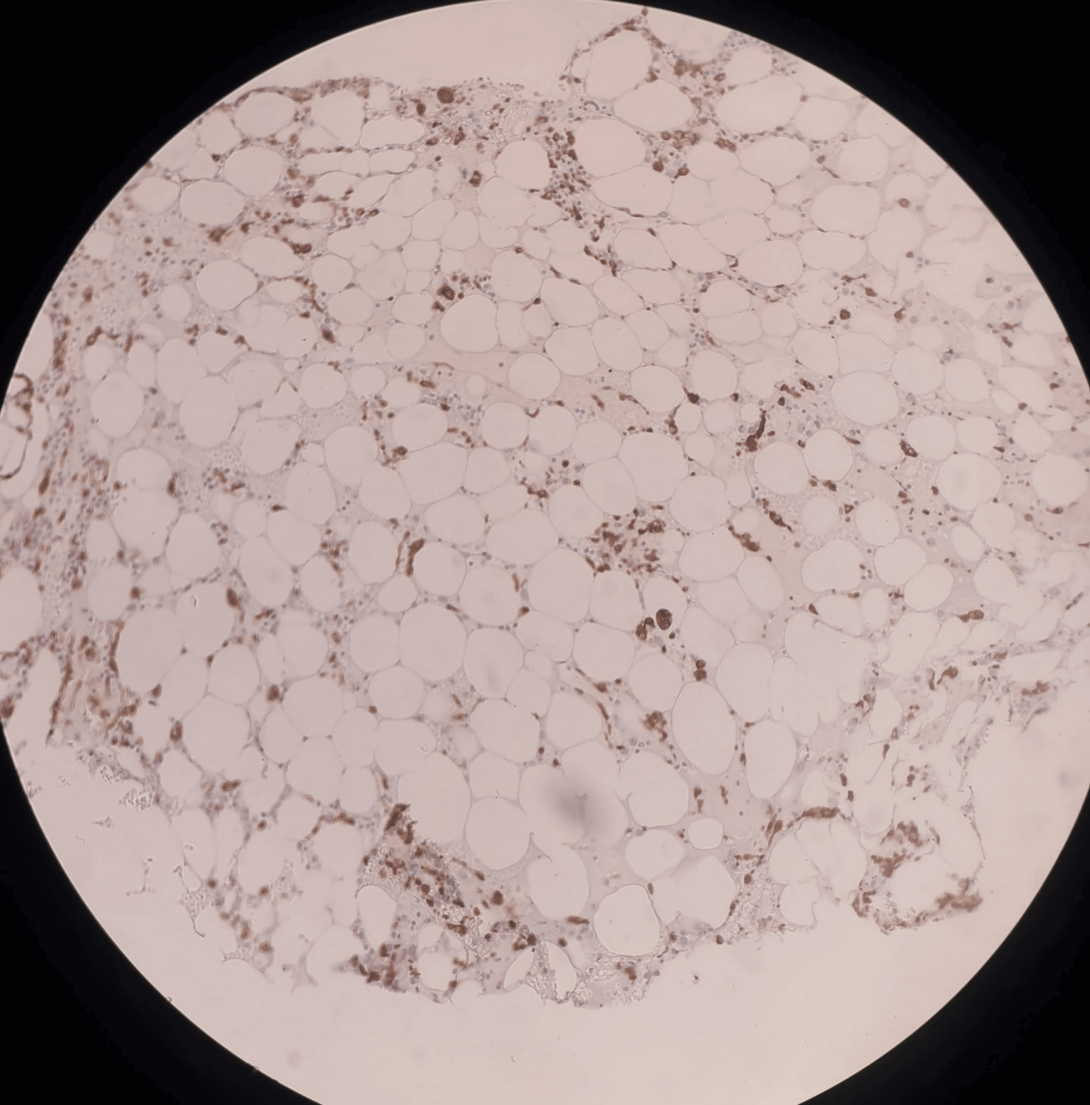
CD68 immunohistochemistry highlighting the prominence of histiocytes (CD68 immunostain, ×200 magnification)

Bone marrow aspiration and biopsy examination revealed hypocellular marrow (10-15% cellularity), suggestive of aplastic anemia (Figures 2-3).

Based on the combination of microangiopathic hemolytic anemia, thrombocytopenia, renal dysfunction, and neurological irritability, the initial differential diagnosis included TTP and aHUS. Therapeutic plasma exchange was initiated according to institutional protocol using 1.5 plasma volumes (60 mL/kg) per session. Daily plasma exchange was performed for five consecutive days without meaningful hematological or clinical improvement.

Treatment was continued, and the patient ultimately received a total of 11 plasma exchange sessions. Despite intensive therapy, no significant response was observed. Eculizumab was subsequently administered along with supportive management. Nevertheless, the patient’s condition progressively deteriorated, and he died following a massive pulmonary hemorrhage.

## Discussion

The clinical presentation was strongly suggestive of TMA, given the coexistence of anemia, thrombocytopenia, renal impairment, and peripheral smear evidence of microangiopathic hemolysis. Accordingly, treatment was initiated with plasma exchange and comprehensive supportive care. The absence of severe ADAMTS13 deficiency made TTP less likely and shifted diagnostic consideration toward aHUS. Persistent thrombocytopenia and ongoing schistocytosis despite repeated plasma exchange further supported this diagnosis.

A particularly unusual finding in this case was the presence of markedly hypocellular bone marrow. Rather than the expected hypercellular marrow typically described in HUS, marrow examination revealed features compatible with aplastic anemia. Because of the lack of response to plasma exchange, additional therapies, including pulse methylprednisolone, intravenous immunoglobulin, and eculizumab, were administered. Despite these interventions, no clinical or hematological recovery occurred.

Hemolytic uremic syndrome is a form of TMA defined by the triad of hemolytic anemia, thrombocytopenia, and acute kidney injury. Both inherited and acquired disturbances in complement regulation may precipitate the disease. Excessive complement activation damages endothelial cells, resulting in vascular swelling, detachment, and microvascular thrombosis. Passage of erythrocytes through these injured vessels causes mechanical fragmentation, producing schistocytes and Coombs-negative hemolytic anemia. Clinically, the kidneys are most frequently affected, although involvement of the central nervous system, heart, lungs, gastrointestinal tract, skeletal muscle, and pancreas may also occur [4,5].

Approximately 60% of patients with genetically mediated forms of HUS eventually progress to end-stage kidney disease. Predisposition to aHUS is commonly inherited in an autosomal dominant pattern with incomplete penetrance. Although familial clustering may occur, many affected individuals have no identifiable family history. Consequently, aHUS should remain an important differential diagnosis in pediatric patients presenting with acute kidney injury and evidence of TMA.

The complement system is a major component of innate immunity and contributes to pathogen elimination, inflammatory signaling, and clearance of damaged cells. Activation occurs through the classical, lectin, and alternative pathways. Dysregulated activation of the alternative pathway is the principal mechanism underlying most cases of aHUS [6-8].

Current therapeutic options for aHUS include plasma exchange, immunosuppressive therapy, corticosteroids, and complement inhibition with eculizumab [9]. In many centers within India, therapeutic plasma exchange remains the initial treatment approach because it removes pathogenic complement-related factors and replenishes deficient regulatory proteins [10]. Patients with anti-CFH antibody-mediated disease often benefit from plasma exchange combined with immunosuppressive agents such as corticosteroids, azathioprine, mycophenolate mofetil, or rituximab. In contrast, eculizumab has become the preferred first-line treatment in many developed healthcare systems. By inhibiting complement component C5 and preventing formation of the membrane attack complex, eculizumab effectively suppresses terminal complement activation and reduces the need for repeated plasma exchange procedures [11].

The coexistence of aHUS and markedly hypocellular marrow observed in our patient is highly unusual and has rarely been described. Unlike the expected clinical course in many patients receiving contemporary therapy, our patient experienced relentless disease progression culminating in death.

## Conclusions

Diagnosing atypical HUS remains difficult because no single laboratory investigation can definitively establish the diagnosis. Complement testing may be inconclusive, and many patients lack detectable genetic or acquired abnormalities involving complement regulation. Consequently, diagnosis relies heavily on clinical assessment and exclusion of alternative causes of TMA.

Early recognition and timely initiation of treatment are essential for improving outcomes. Clinicians should maintain a high index of suspicion for aHUS in all patients presenting with TMA. The occurrence of aHUS with hypocellular bone marrow appears exceptionally rare and may be associated with an adverse prognosis. Further studies are required to clarify the biological relationship between complement-mediated TMA and bone marrow failure.
